# Mobilizing stem cells and inhibition of autoimmunity synergistically modify autoimmune disease to relieve pain: a potential MRG-001 therapy for rheumatoid arthritis

**DOI:** 10.1097/js9.0000000000005353

**Published:** 2026-05-07

**Authors:** Weixin Zhang, Francisco Lopez, Mei Wan, Junying Zheng, Russell Wesson, Zhaoli Sun, Xu Cao

**Affiliations:** a Department of Orthopedic Surgery, Johns Hopkins University School of Medicine, Baltimore, Maryland, USA; b Department of Surgery, Johns Hopkins University School of Medicine, Baltimore, Maryland, USA

**Keywords:** immunomodulation, joint protection, pain relief, rheumatoid arthritis, synergistic therapy

## Abstract

**Objectives:**

Rheumatoid arthritis (RA) is a chronic autoimmune disease characterized by persistent synovial inflammation, bone erosion, cartilage destruction, and debilitating pain. Current therapies often fall short in preventing structural damage and restoring immune balance. To address these limitations, we evaluated MRG-001 – a fixed-dose combination of plerixafor (AMD3100) and low-dose tacrolimus (FK506) with reported immunoregulatory and regenerative potential – in preclinical models of RA.

**Methods:**

The therapeutic efficacy of MRG-001 was assessed in collagen-induced arthritis and collagen antibody-induced arthritis mouse models. Mice received subcutaneous MRG-001, and clinical, histological, molecular, and behavioral endpoints were evaluated to examine inflammation, immune modulation, bone integrity, angiogenesis, and pain.

**Results:**

MRG-001 treatment significantly reduced joint swelling, arthritis scores, and pro-inflammatory cytokine expression (TNF-α, IL-6, IL-17a) in both models. It was associated with mobilization of CD45^−^Sca1^+^CD29^+^ mesenchymal stem cells, increased CD4^+^CD25^+^Foxp3^+^ regulatory T cells, and reduced CD4^+^RORγt^+^ Th17 cells in peripheral blood. MRG-001 preserved cartilage integrity was associated with reduced osteoclast-mediated bone erosion, and improved trabecular bone structure. It was associated with reduced fibroblast-like synoviocyte activation, lower TGF-β signaling activity, and decreased RANKL expression in joint tissues. Additionally, MRG-001 was associated with reduced pathological angiogenesis (CD31^+^EMCN^+^ vessels) and sensory nerve innervation (PGP9.5^+^, CGRP^+^ fibers), corresponding with improved pain-like behaviors and physical function.

**Conclusions:**

MRG-001 exerts broad therapeutic effects in RA by modulating immune responses, preserving joint architecture, and alleviating pain. These findings support its potential as a novel, multi-modal disease-modifying therapy for RA and warrant further clinical investigation.

## Introduction

Rheumatoid arthritis (RA) is a chronic, systemic autoimmune disease marked by persistent synovial inflammation, progressive cartilage degradation, and bone erosion, ultimately resulting in joint deformity, functional disability, and reduced quality of life^[1–4]^. Affecting approximately 0.5–1% of the global population^[5]^, RA imposes a substantial healthcare burden due to its chronic course, associated comorbidities, and high treatment costs. While conventional synthetic and biologic disease-modifying antirheumatic drugs (DMARDs) have improved disease control, many patients fail to achieve sustained remission or suffer from treatment-related adverse effects^[6–8]^. This underscores the urgent need for novel therapies that can simultaneously target inflammation, tissue damage, and pain.

Emerging evidence identifies immune dysregulation, fibroblast-like synoviocyte (FLS) activation, and pathological neurovascular remodeling as key drivers of RA pathogenesis^[9–11]^. Transforming Growth Factor Beta (TGF-β) signaling plays a complex, context-dependent role, functioning as both an immunoregulatory and pathogenic factor. TGF-β1 is upregulated in the synovium of RA patients – including in synovial lining cells, macrophages, and endothelial cells – and is elevated in synovial fluid compared to osteoarthritis and healthy controls^[12–14]^. In RA, TGF-β promotes Th17 cell differentiation in the presence of IL-6, contributes to synovial fibrosis and hyperplasia, and enhances pathological angiogenesis^[15–17]^. In conjunction with inflammatory cytokines such as TNF-α, IL-1β, and IL-6, TGF-β upregulates receptor activator of NF-κB ligand (RANKL) in FLSs, promoting osteoclastogenesis and bone erosion^[18,19]^. The imbalance between pro-inflammatory Th17 cells and regulatory T cells (Tregs), alongside elevated TNF-α and IL-17A, further perpetuates chronic inflammation and joint destruction^[8,20]^. Additionally, aberrant angiogenesis and sensory nerve innervation within the synovial microenvironment exacerbate joint pain. Targeting the TGF-β pathway and its downstream mediators (e.g., SMADs) offers therapeutic potential but requires selective modulation to preserve essential immunoregulatory functions, such as Treg maintenance.

MRG-001 is a fixed-dose combination therapy consisting of AMD3100 and low-dose FK506, developed for subcutaneous administration based on discoveries by Dr. Sun’s laboratory at Johns Hopkins Medicine^[21–29]^. Originally investigated in the context of liver allograft tolerance, MRG-001 was found to synergistically mobilize bone marrow-derived stem cells and regulatory T cells when AMD3100 and low-dose FK506 were co-administered subcutaneously^[22]^. AMD3100, a CXCR4 antagonist, is FDA-approved for mobilizing CD34^+^ hematopoietic stem cells in patients with lymphoma or multiple myeloma^[30]^, while FK506 is an immunosuppressant commonly used at higher doses to prevent allograft rejection. In MRG-001, FK506 is used at a substantially lower, non-immunosuppressive dose.

Preclinical studies have demonstrated that this combination (also referred to as AF) induces long-term allograft survival without continuous immunosuppression by promoting allograft chimerism^[22,24,29,31–33]^. In various models, MRG-001 has also been shown to enhance wound healing, reduce fibrosis, accelerate liver regeneration^[26]^, prevent postoperative adhesions^[27]^, and attenuate intestinal inflammation. These findings highlight its dual anti-inflammatory and regenerative properties across diverse settings of tissue injury and immune activation. A recent Phase I clinical trial confirmed the favorable safety profile of MRG-001 and demonstrated its ability to mobilize stem and immunoregulatory cells while downregulating 31 inflammation-related pathways, including TGF-β, TNF-α/NF-κB, and IL-6/JAK/STAT3 signaling^[34]^. These data support its potential as a multi-target therapeutic approach for diseases driven by immune dysregulation and tissue injury, such as RA. However, its efficacy in treating the structural and symptomatic manifestations of RA remains to be fully established.

In this study, we evaluate the therapeutic effects of MRG-001 in two preclinical models of RA: collagen-induced arthritis (CIA) and collagen antibody-induced arthritis (CAIA). We demonstrate that MRG-001 significantly reduces synovial inflammation, restores immune homeostasis, suppresses Th17 differentiation, and mobilizes CD45^−^Sca1^+^CD29^+^ mesenchymal stem cells (MSCs). Moreover, it protects against cartilage and bone destruction, inhibits pathological synovial angiogenesis and innervation, and alleviates pain-like behaviors. These findings provide compelling preclinical evidence supporting MRG-001 as a novel, multi-modal therapeutic strategy for RA.

## Material and methods

### Study design

This study evaluated the therapeutic efficacy of MRG-001 in joint protection and pain relief using two clinically relevant mouse models of RA: the CIA model and the CAIA model. The CIA model was established by subcutaneous immunization with chicken type II collagen (100 μg) emulsion, administered at the base of the tail, followed by a booster immunization on day 21^[35–37]^. The CAIA model was induced via intraperitoneal injection of a monoclonal antibody cocktail targeting type II collagen, followed by lipopolysaccharide (LPS; 25 μg) administration on day 3 to accelerate disease onset.

Mice in both CIA and CAIA models were randomly assigned to receive either saline or MRG-001 treatment (1 μL/g body weight; AMD3100: 2.4 μg/g, FK506: 0.1 μg/g) via subcutaneous injection every other day, starting from day 4 post-antibody injection in CAIA mice, or from day 22 in CIA mice, for up to 6 weeks. Age-matched, non-immunized mice were used as healthy controls (DBA-Veh). At the study endpoint, mice were euthanized using an overdose of isoflurane and perfused with phosphate-buffered saline (PBS), with or without 4% paraformaldehyde (PFA), depending on downstream analyses. Tissues were collected for histological, molecular, and imaging studies.

A total of 135 mice were used in this study: 20 mice in a pilot, blinded study and 115 mice in subsequent open-label studies across both models. All animal procedures were approved by the Institutional Animal Care and Use Committee (IACUC) of The Johns Hopkins University School of Medicine (protocol number MO21M276). Mice were housed in the animal facility of The Johns Hopkins University School of Medicine. All experiments were conducted in accordance with institutional and national guidelines for the care and use of laboratory animals, and results are reported in compliance with the ARRIVE guidelines (Animals in Research: Reporting In Vivo Experiments)^[38]^.

### Mouse models of RA

Female DBA/1J mice (6 weeks old, strain number: 000670) were purchased from The Jackson Laboratory. All animal procedures were approved by the Johns Hopkins Animal Care and Use Committee (ACUC).

The CIA model was established by subcutaneous immunization with chicken type II collagen (#20012, Chondrex) emulsified in Complete Freund’s Adjuvant (#7001, Chondrex). The emulsion was prepared according to the manufacturer’s instructions by homogenizing the components at 15,000 rpm. Each mouse received 100 μg of the emulsion via subcutaneous injection into the tail. A booster immunization was administered on day 21 with 100 μg of chicken type II collagen emulsified in Incomplete Freund’s Adjuvant (#7002, Chondrex)^[35]^.

The CAIA model was induced by intraperitoneal injection of a monoclonal antibody cocktail targeting type II collagen (#53100, 1.5 mg/mouse), followed by an intraperitoneal injection of lipopolysaccharide (#53100, LPS, 25 μg) on day 3 to accelerate disease progression^[37]^.

### MicroCT scanning and analysis

Joint tissues were harvested immediately following euthanasia, fixed in 4% PFA for 48 h, and then transferred to PBS for storage. High-resolution micro-computed tomography (μCT) imaging was performed using the Skyscan 1172 system. Scanning parameters were set at 55 kVp voltage, 163 μA current, and a pixel resolution of 6.0 μm^[36]^.

Image reconstruction and quantitative analysis were conducted using NRecon v1.6 and CTAn v1.9 software (Skyscan). Sagittal-plane sections of the knee joints were used for three-dimensional histomorphometric analysis of subchondral bone. Structural parameters evaluated included trabecular bone volume per tissue volume (BV/TV), trabecular number (Tb.N), trabecular separation (Tb.Sp), and trabecular pattern factor (Tb.Pf)^[39]^.

For visualization, five sequential sagittal images were selected to generate three-dimensional reconstructions of the subchondral bone using CTVol v2.0 (Skyscan), providing detailed anatomical assessment of knee joint architecture.

### Hargreaves test

The Hargreaves test was used to evaluate thermal nociceptive thresholds in mice. Prior to testing, animals were acclimated to the testing environment for at least 1 h. After acclimation, a focused radiant heat source generated by a light beam (IITC Life Science Inc.) was applied to the plantar surface of each hind paw. The intensity of the heat stimulus was set at 25% (approximately 62.5 °C at the glass source).

The latency to paw withdrawal was recorded as the response time, representing the animal’s sensitivity to thermal stimuli. Each mouse was tested at least three times, with sufficient intervals between trials to prevent sensitization. The average latency from the three trials was calculated for each animal and used for subsequent analysis. This standardized protocol allowed for consistent and reproducible assessment of thermal hyperalgesia^[40]^.

### Von Frey test

The Von Frey test was used to assess mechanical sensitivity in mice. Prior to testing, mice were placed individually on an elevated metal mesh platform within transparent plastic enclosures and acclimated for at least 1 h. A calibrated Von Frey filament applying 0.4 g of force (BIOSEB) was applied to the plantar surface of each hind paw. The filament was applied for up to 3 s or until a withdrawal response was observed.

Paw withdrawal frequency was recorded over 10 consecutive stimulations, with a 2-s interval between each application. Each mouse was tested at least three times, and the average withdrawal frequency was calculated for subsequent analysis. This method provided a reliable measure of mechanical allodynia^[41]^.

### Active wheel test

Spontaneous wheel-running activity was evaluated using specialized activity wheels (Model BIO-ACTIVW-M, Bioseb) equipped with software for continuous monitoring and data acquisition. Mice were acclimated to the wheel cage environment overnight before testing. During the testing period, mice were housed under a 12-h light/dark cycle and monitored continuously for 48 h.

Running distance and speed were automatically recorded and analyzed using the manufacturer’s software, providing a quantitative assessment of voluntary locomotor activity and physical function^[41,42]^.

### qPCR

Total RNA was extracted from tissue samples using the RNeasy Mini Kit (Qiagen) according to the manufacturer’s protocol. RNA purity was assessed by measuring the 260/280 nm absorbance ratio. Complementary DNA (cDNA) was synthesized from total RNA using the PrimeScript RT Master Mix (Takara) following the manufacturer’s instructions.

Quantitative PCR (qPCR) was performed using SYBR Green Master Mix (Qiagen, Hilden, Germany) on a C1000 Thermal Cycler (Bio-Rad Laboratories, Hercules, USA). Relative gene expression was calculated using the 2^–ΔΔCT method, with Gapdh serving as the internal control for normalization. Primer sequences used for amplification were as follows:

GAPDH forward: 5′-CATCACTGCCACCCAGAAGACTG-3′

GAPDH reverse: 5′-ATGCCAGTGAGCTTCCCGTTCAG-3′

TNF-α forward: 5′-CCCTCACACTCAGATCATCTTCT-3′

TNF-α reverse: 5′-GCTACGACGTGGGCTACAG-3′

IL-17A forward: 5′-TCAGCGTGTCCAAACACTGAG-3′

IL-17A reverse: 5′-CGCCAAGGGAGTTAAAGACTT-3′

IL-10 forward: 5′-CTTACTGACTGGCATGAGGATCA-3′

IL-10 reverse: 5′-GCAGCTCTAGGAGCATGTGG-3′

IL-6 forward: 5′-TAGTCCTTCCTACCCCAATTTCC-3′

IL-6 reverse: 5′-TTGGTCCTTAGCCACTCCTTC-3′

Primers were selected from the PrimerBank database and validated in previous studies. Specificity was confirmed using NCBI BLAST to rule out off-target binding. A single sharp peak in the melt curve analysis confirmed amplification specificity, and primer linearity was validated through standard curves generated from serial 10-fold dilutions of cDNA templates.

### Flow cytometry

Flow cytometry was performed on peripheral blood and bone marrow cells collected from CIA mice treated with saline, FK506, AMD3100, or MRG-001, as well as from non-immunized DBA/1J mice treated with saline.

Peripheral blood was collected from the right ventricle using K2-EDTA blood collection tubes (BD Vacutainer) and processed by mixing with an equal volume of Ammonium–Chloride–Potassium (ACK) lysis buffer (Quality Biological, Inc., Gaithersburg, MD). Samples were centrifuged at 350 × g for 5 min at 4°C to lyse red blood cells.

Bone marrow was harvested from femurs and tibias of the hind limbs by flushing the marrow cavities with FACS buffer (PBS containing 5% fetal bovine serum, FBS). The resulting cell suspension was gently pipetted to obtain a single-cell suspension and filtered through a 40-μm cell strainer to remove debris. Red blood cells were lysed using ACK buffer for 10 min, followed by washing and resuspension in 100 μL of FACS buffer.

Cells were incubated with fluorochrome-conjugated antibodies for 30 min at 4°C in the dark. The following antibodies were used (all at 1:100 dilution unless otherwise specified):

Alexa Fluor 647-conjugated anti-mouse Sca1 (Ly-6A/E) (565355, BD Biosciences)

Pacific Blue-conjugated anti-mouse CD29 (102224, BioLegend)

APC-Cy7-conjugated anti-mouse CD45 (557659, BD Biosciences)

BUV395-conjugated anti-mouse CD4 (563790, BD Biosciences)

PE-CF594-conjugated anti-mouse RORγt (562684, BD Biosciences)

BB515-conjugated anti-mouse CD25 (564424, BD Biosciences)

Alexa Fluor 647-conjugated anti-mouse FoxP3 (560401, BD Biosciences)

Data were acquired on a BD LSR II flow cytometer (BD Biosciences) and analyzed using FlowJo software (version 10, BD Biosciences).

### Immunofluorescence and immunohistochemical staining

Knee joint tissues were collected immediately after euthanasia and fixed in 4% PFA for 48 h. Following fixation, samples were decalcified in 10% ethylenediaminetetraacetic acid (EDTA, pH 7.4) at room temperature for 2 weeks, then dehydrated and embedded either in paraffin or optimal cutting temperature (OCT) compound (Sakura Finetek).

Sagittal sections (4 μm thick) from paraffin-embedded joints were processed for immunohistochemical (IHC) staining, tartrate-resistant acid phosphatase (TRAP) staining (per manufacturer’s protocol), and Safranin O Fast Green staining following standard histological protocols. Sagittal sections (40 μm thick) from OCT-embedded joints were used for immunofluorescence (IF) staining. Sections were incubated overnight or for up to 48 h at 4°C with the following primary antibodies:

CGRP (1:200, ab36001)

CD31 (1:100, 14-0311-82)

Endomucin (1:200, sc-65495)

pSmad2 (1:100, 44-244G)

Vimentin (1:100, sc-6260)

RANKL (1:200, 23408-1-AP)

COX-2 (1:200, 12282)

For IF staining, sections were subsequently incubated with appropriate fluorescent secondary antibodies and DAPI (1:250, H-1200, Vector) for 1 h at room temperature in the dark. For IHC staining, sections were treated with 0.06% hydrogen peroxide for 10 min to quench endogenous peroxidase activity prior to secondary antibody incubation. Detection was performed using a horseradish peroxidase-streptavidin system (Dako), followed by hematoxylin counterstaining (Dako, S3309).

Imaging was performed using either an Olympus BX51 microscope with a DP71 camera or a Zeiss LSM 880 confocal microscope. Image analysis was conducted using ImageJ software (National Institutes of Health, Bethesda, MD).

### Whole-mount PGP9.5 staining of knee joints

Following fixation and decalcification in 10% EDTA (pH 7.4) for 2 weeks at room temperature, knee joints were bisected along the sagittal plane. Samples were then delipidated and dehydrated through a graded methanol series and subjected to an antigen retrieval protocol involving collagenase A and boric acid, as previously described^[42]^.

Tissues were incubated with primary antibody against PGP9.5 (1:200, SAB4503057) for 72 h at 37°C, followed by incubation with appropriate fluorescent secondary antibodies under the same conditions. Samples were optically cleared using dibenzyl ether.

Imaging was performed with a Keyence BZ-X710 microscope, and image analysis was conducted using ImageJ software.

### Statistics

Statistical analyses were performed using GraphPad Prism version 10.0 (Boston, MA, USA). Data are presented as mean ± standard deviation. Comparisons among multiple groups were conducted using one-way analysis of variance (ANOVA) followed by Tukey’s post hoc test for multiple comparisons. For comparisons between two groups, an unpaired, two-tailed Student’s t-test was used. A P-value of less than 0.05 was considered statistically significant.

## Results

### MRG-001 treatment is associated with increased CD45^−^Sca1^+^CD29^+^ MSCs, and normalization of CD4^+^RORγt^+^ Th17 cells, CD4^+^Foxp3^+^ regulatory T cells in the peripheral blood of CIA mice

**Figure 1.**
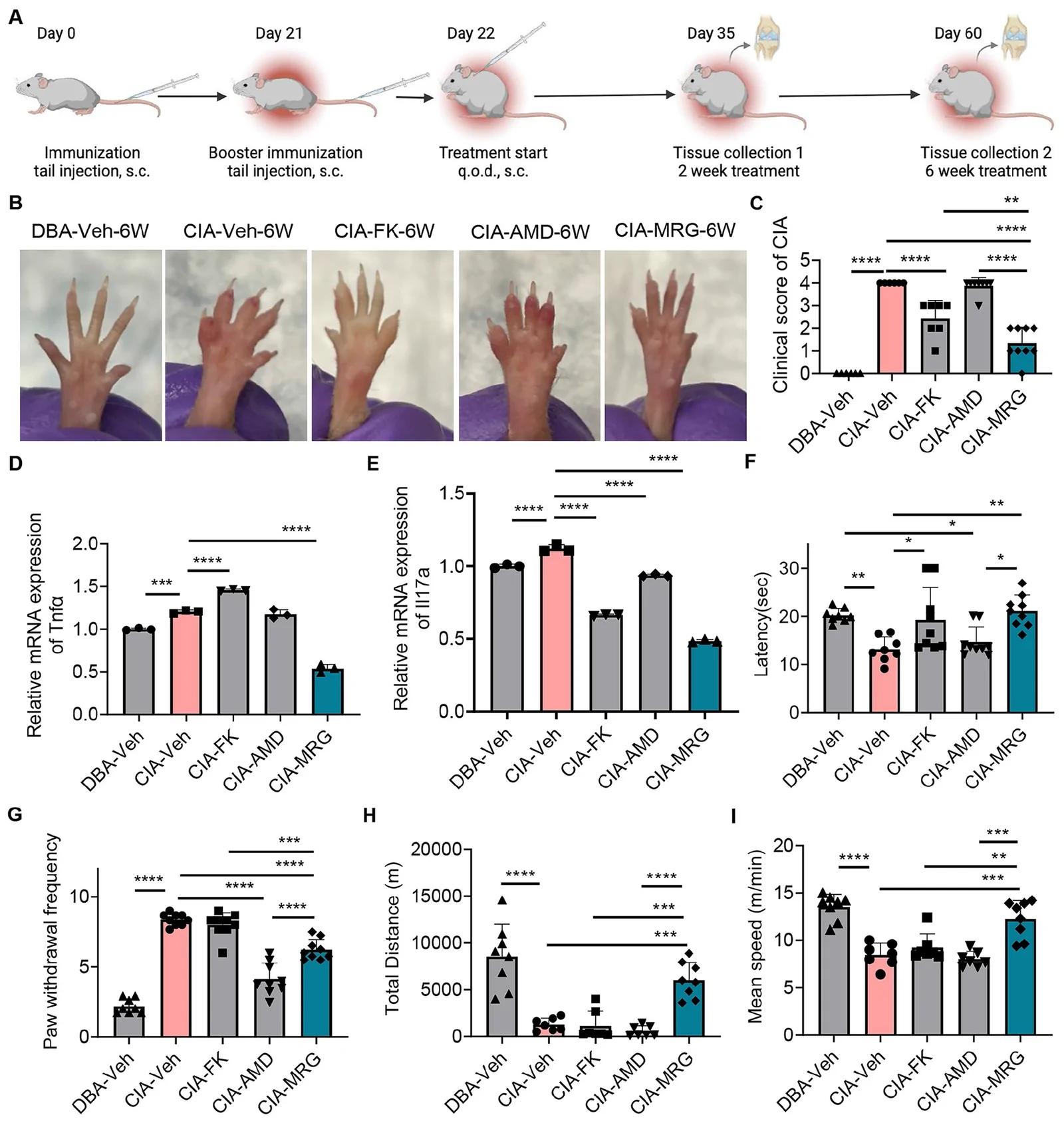
MRG-001 treatment alleviates paw inflammation, downregulates TNF-α and IL-17A expression, and reduces pain-like behaviors in CIA mice. (A) Schematic diagram of the experimental timeline. (B) Representative images of hind paws from CIA mice treated with vehicle, FK506, AMD3100, or MRG-001, and from non-immunized DBA/1J healthy controls treated with vehicle for 6 weeks. (C) Clinical scoring of paw inflammation severity in CIA mice across treatment groups and in DBA/1J healthy controls (*n* ≥ 6; one-way ANOVA with Tukey’s multiple comparisons test). (D, E) Quantitative PCR analysis of Tnfα (D) and Il17a (E) gene expression in knee joint tissues of CIA mice and healthy controls after 6 weeks of treatment (*n* = 3; one-way ANOVA with Tukey’s multiple comparisons test). (F, G) Behavioral assessment of thermal hypersensitivity via paw withdrawal latency (F) and mechanical hypersensitivity via paw withdrawal frequency (G) in DBA/ 1J and CIA mice treated for 6 weeks (*n* ≥ 8; one-way ANOVA with Tukey’s multiple comparisons test). (H, I) Spontaneous activity measured using activity wheels: total distance traveled (H) and mean running speed (I) in CIA mice and DBA/1J controls after 6 weeks of treatment (*n* ≥ 6; one-way ANOVA with Tukey’s multiple comparisons test). *P<0.05, **P<0.01, ***P<0.005, ****P<0.001.

To evaluate the therapeutic efficacy of MRG-001 in RA, we established a CIA model in mice following our previously reported protocol^[36]^. Immunized mice were randomly assigned to four treatment groups after booster immunization on day 21 and received placebo (saline), single-agent AMD3100 or FK506, or MRG-001 starting on day 22, administered every other day through day 60 (6-week treatment) (Fig. 1A).

MRG-001 treatment significantly increased the percentage of CD45^−^Sca1^+^CD29^+^ MSCs in peripheral blood, suggesting a potential mobilization effect (Supplemental Digital Content Figure 1A, available at: http://links.lww.com/JS9/H347). On day 35 following immunization, vehicle-treated mice showed elevated frequencies of both CD4^+^RORγt^+^ Th17 cells and CD4^+^Foxp3^+^ regulatory T cells (Tregs) compared to non-immunized controls (Supplemental Digital Content Figure 1B and C, available at: http://links.lww.com/JS9/H347). Notably, a 2-week course of MRG-001 was associated with a reduced frequency of CD4^+^RORγt^+^ Th17 cells (Supplemental Digital Content Figure 1B and C, available at: http://links.lww.com/JS9/H347). By day 60, the frequencies of both Th17 and Treg cells remained elevated in immunized mice compared to non-immunized DBA controls. However, MRG-001 treatment resulted in Th17 and Treg cell frequencies that approached levels observed in non-immunized control mice (Supplemental Digital Content Figure 1D and E, available at: http://links.lww.com/JS9/H347). These findings suggest that MRG-001 is associated with increased circulating MSCs and modulation of Th17/Treg balance in the peripheral blood of CIA mice.

### MRG-001 treatment alleviates paw inflammation and reduces pain-like behaviors in CIA mice

To assess the therapeutic efficacy of a 6-week course of MRG-001 in RA, clinical severity of arthritis in the hind paws of CIA mice was evaluated using previously established scoring criteria^[35]^. Saline (vehicle)- and AMD3100-treated mice exhibited pronounced swelling and erythema in all digits of their hind paws, while low-dose FK506-treated mice showed these signs in only some digits. Remarkably, MRG-001-treated mice exhibited minimal inflammatory signs after 6 weeks of treatment, as assessed on day 60 (Fig. 1B). Correspondingly, clinical arthritis scores reached an average of 4 in saline- and AMD3100-treated mice, were reduced to approximately 2.5 with low-dose FK506 and were lowest (~1.5) in MRG-001-treated mice, suggesting a potential anti-inflammatory benefit (Fig. 1C).

To further evaluate the anti-inflammatory activity of MRG-001, mRNA expression levels of pro-inflammatory cytokines TNF-α and IL-17a were measured by qPCR in joint tissues. Compared to non-immunized DBA mice (DBA-Veh), TNF-α expression was significantly elevated in vehicle-, AMD3100-, and FK506-treated CIA mice, but was markedly reduced in MRG-001-treated mice (Fig. 1D). Similarly, IL-17a expression was increased in saline-treated CIA mice, moderately reduced by AMD3100 and FK506, and suppressed to the lowest level in MRG-001-treated mice (Fig. 1E). These results suggest that MRG-001 treatment is associated with reduced expression of pro-inflammatory cytokines in the CIA model.

Given that RA severity correlates with increased pain and reduced mobility, we next evaluated pain-like behaviors in CIA mice. Thermal hyperalgesia, assessed by the Hargreaves test, showed that paw withdrawal latency decreased from ~20 s in non-immunized mice to ~13 s in saline- and AMD3100-treated CIA mice. Low-dose FK506 partially improved latency to ~18 s, whereas MRG-001 treatment restored latency to normal levels (~20 s), suggesting a reversal of thermal hypersensitivity (Fig. 1F). Mechanical hyperalgesia was assessed using the Von Frey test. CIA mice exhibited significantly increased paw withdrawal frequency compared to non-immunized controls. While FK506 did not significantly alter this response, MRG-001 and AMD3100 significantly reduced paw withdrawal frequency, suggesting a partial mitigation of mechanical pain sensitivity (Fig. 1G).

To assess functional improvement, voluntary locomotion was measured using a running wheel. Over a 2-day period, non-immunized DBA mice demonstrated significantly greater running speed and distance compared to CIA mice treated with saline, FK506, or AMD3100. Importantly, MRG-001-treated CIA mice exhibited travel speed (Fig. 1H) and distance (Fig. 1I) comparable to healthy controls, indicating restoration of joint function and pain relief. Taken together, while singleagent FK506 or AMD3100 conferred partial benefits, MRG-001, a fixed-dose combination of both agents, was associated with greater improvements in inflammation, pain-related behaviors, and joint function in CIA mice, supporting its potential therapeutic utility.

### MRG-001 treatment ameliorates cartilage destruction, prevents bone erosion, and improves bone microarchitecture in CIA mice

**Figure 2.**
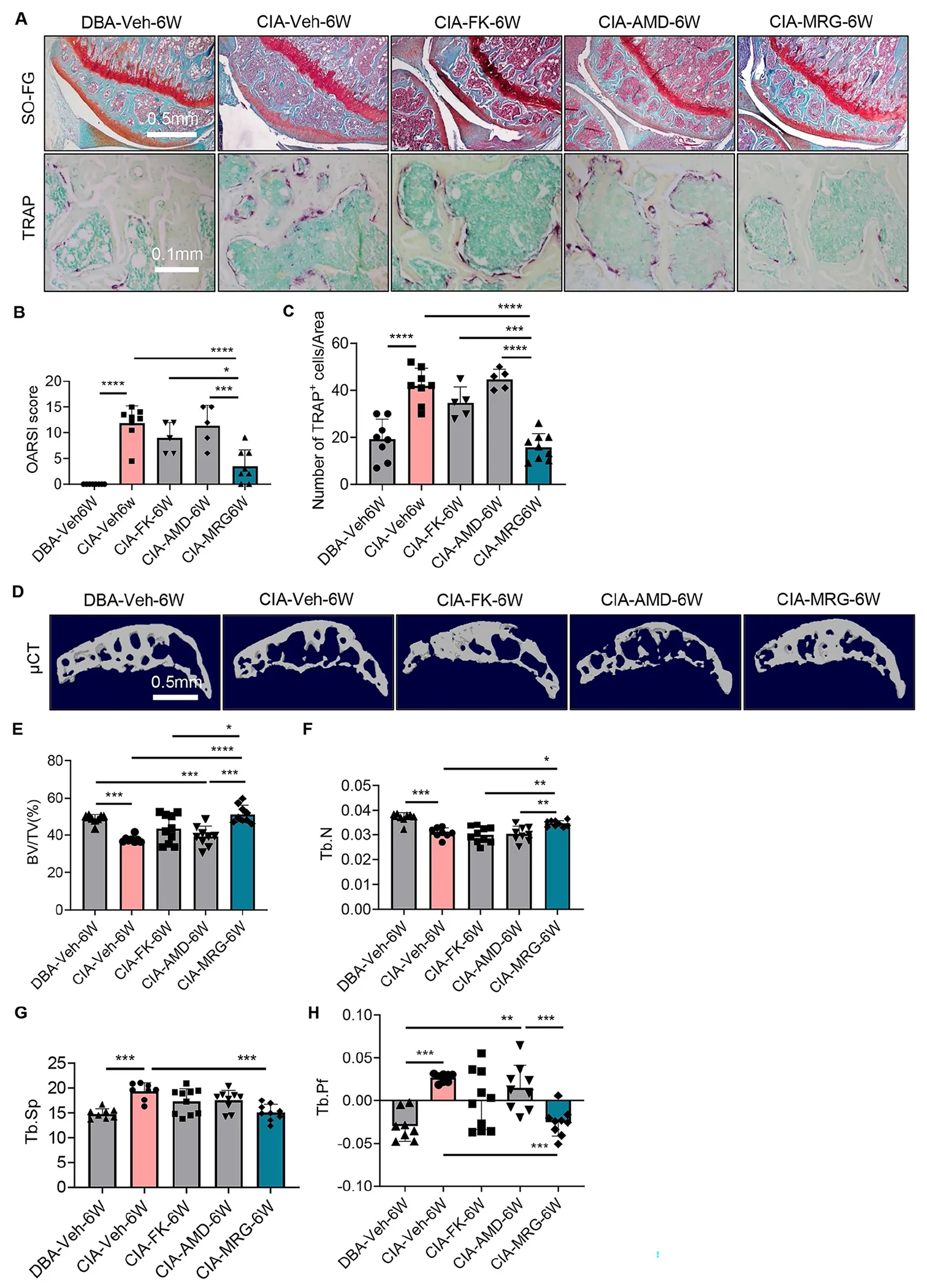
MRG-001 treatment ameliorates cartilage destruction and bone erosion in CIA mice. (A) Representative images of Safranin O–Fast Green (SO-FG) staining (upper row) and TRAP staining (lower row) of knee joint sections from DBA/1J healthy controls and CIA mice treated with vehicle, FK506, AMD3100, or MRG-001 for 6 weeks. (B, C) Quantitative analysis of SO-FG staining (B) and TRAP-positive cell counts (C) in knee joint sections from the same groups (*n* ≥ 5; one-way ANOVA with Tukey’s multiple comparisons test). (D) Representative three-dimensional microCT images (sagittal view) of the subchondral bone in the medial tibial compartment from DBA/1J and CIA mice treated as above. (E–H) Quantitative microCT analysis of subchondral bone parameters, including bone volume per tissue volume (BV/TV) (E), trabecular number (Tb.N) (F), trabecular separation (Tb.Sp) (G), and trabecular pattern factor (Tb.Pf) (H) in DBA/1J and CIA mice after 6 weeks of treatment (*n* ≥ 8; one-way ANOVA with Tukey’s multiple comparisons test). *P<0.05, **P<0.01, ***P<0.005, ****P<0.001.

Cartilage and bone damage are hallmarks of RA and contribute significantly to joint dysfunction. To assess the protective effects of MRG-001 on joint integrity, we evaluated cartilage preservation and bone microarchitecture in CIA mice. Safranin O–Fast Green (SO-FG) staining (Fig. 2A, upper panels), a histological method used to assess cartilage integrity, revealed substantial loss of proteoglycans (red to orange staining) in the cartilage of CIA mice treated with saline, AMD3100, or low-dose FK506, indicating cartilage degradation. In contrast, MRG-001-treated mice exhibited proteoglycan content comparable to healthy controls (DBA-Veh), suggesting a potential association with reduced cartilage degradation.

To assess bone resorption, we performed TRAP staining, which identifies osteoclasts – cells responsible for bone resorption. TRAP^+^ osteoclasts were significantly increased in the subchondral bone of saline-, AMD3100-, and FK506-treated CIA mice compared to healthy controls (Fig. 2A, lower panels). In contrast, TRAP^+^ cell counts in MRG-001-treated mice were similar to those in non-immunized controls, suggesting reduced osteoclast activity associated with treatment (Fig. 2B), indicating reduced bone resorption. We next quantified joint damage using the Osteoarthritis Research Society International (OARSI) scoring system, which assesses the severity and extent of cartilage degeneration^[43]^. OARSI scores were significantly elevated in CIA mice treated with saline or single agents at 6 weeks post-treatment, while MRG-001-treated mice exhibited lower scores, approaching those of healthy controls, indicating a potential improvement in cartilage integrity (Fig. 2C). Micro-computed tomography (micro-CT) was used to evaluate bone volume and architecture. CIA mice treated with saline or AMD3100 exhibited significant reductions in bone volume compared to nonimmunized controls (Fig. 2D and E). FK506 modestly increased bone volume, while MRG-001 was associated with bone volume measures approaching those of healthy controls (Fig. 2E).

To further assess bone quality, we measured key trabecular bone parameters, including trabecular number (Tb.N), trabecular separation (Tb.Sp), and trabecular pattern factor (Tb.Pf). At day 35 (2 weeks post-treatment), no significant differences were observed in Tb.N, Tb.Sp, or Tb.Pf among groups, except for trabecular thickness (Tb.Th) (Supplemental Digital Content Figure 2, available at: http://links.lww.com/JS9/H347). By day 60 (6 weeks post-treatment), saline- and single-agent-treated CIA mice exhibited significantly reduced Tb.N, increased Tb.Sp, and elevated Tb.Pf (Fig. 2F–H), indicating deterioration in trabecular structure. Notably, MRG-001-treated mice showed trabecular parameters more similar to healthy controls, suggesting an association with improved bone microarchitecture. Collectively, these findings suggest that MRG-001 may help mitigate cartilage damage and bone erosion, and may contribute to preserving bone structure and joint integrity in CIA mice.

### MRG-001 treatment is associated with reduced FLS activation, lower TGF-β signaling activity, and decreased RANKL expression in CIA mice

**Figure 3.**
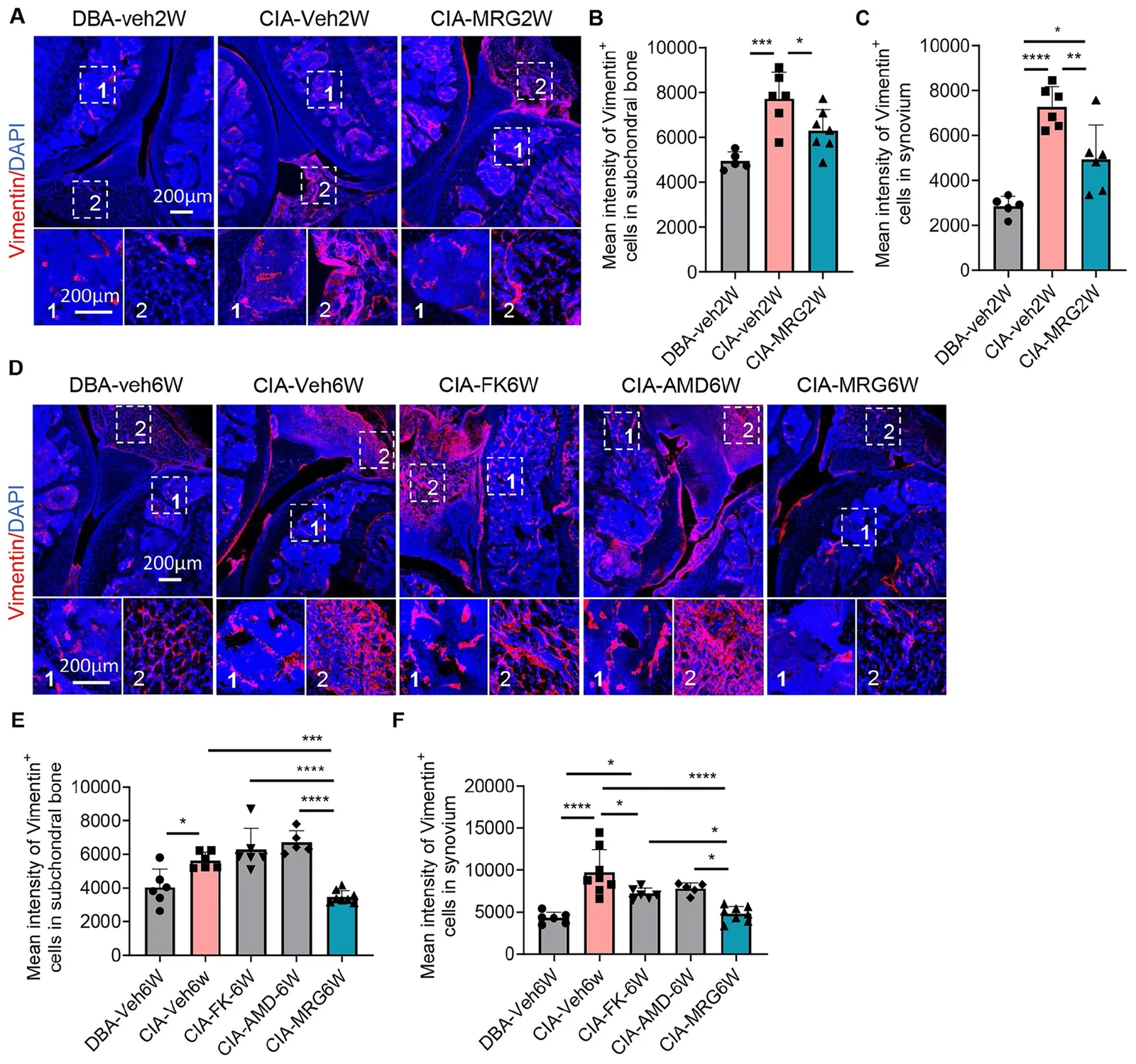
MRG-001 treatment suppresses fibroblast-like synoviocyte differentiation in the knee joints of CIA mice. (A–C) Representative images of vimentin immunofluorescence staining in the tibial subchondral bone and synovium regions (A), and quantitative analysis of vimentin^+^ cells in the subchondral bone (B) and synovium (C) of DBA/1J mice and CIA mice treated with vehicle or MRG-001 for 2 weeks (*n* ≥ 5; one-way ANOVA with Tukey’s multiple comparisons test). (D–F) Representative images of vimentin immunofluorescence staining in the tibial subchondral bone and synovium regions (D), and quantitative analysis of Vimentin-positive cells in the subchondral bone (E) and synovium (F) of DBA/1J mice and CIA mice treated with vehicle, FK506, AMD3100, or MRG-001 for 6 weeks (*n* ≥ 5; one-way ANOVA with Tukey’s multiple comparisons test). *P<0.05; **P<0.01, ***P<0.005, ****P<0.001.

FLSs, a specialized type of fibroblast residing in the synovium, play a central role in the pathogenesis of joint damage and bone erosion in RA^[9,10,44]^. Activated by a complex interplay of immune cells and pro-inflammatory cytokines, FLSs produce key pathogenic mediators including TGF-β and RANKL (Receptor Activator of Nuclear Factor κB Ligand)^[45–47]^. FLSs express canonical fibroblast markers such as vimentin^[48,49]^; therefore, we assessed the presence of vimentin^+^ FLSs in the joints of CIA mice. Fewer vimentin^+^ FLSs were detected in the joints of non-immunized DBA-Veh mice (Fig. 3A–C). In contrast, saline-treated CIA mice exhibited a marked increase in vimentin^+^ FLSs in the subchondral bone and synovium. This increase was attenuated following 2 weeks of MRG-001 treatment. By 6 weeks post-treatment, the number of vimentin^+^ FLSs remained elevated in saline- and single-agent treated CIA mice, while MRG-001-treated mice exhibited fewer vimentin^+^ FLSs compared to other groups in both subchondral bone and synovium (Fig. 3D–F).

**Figure 4.**
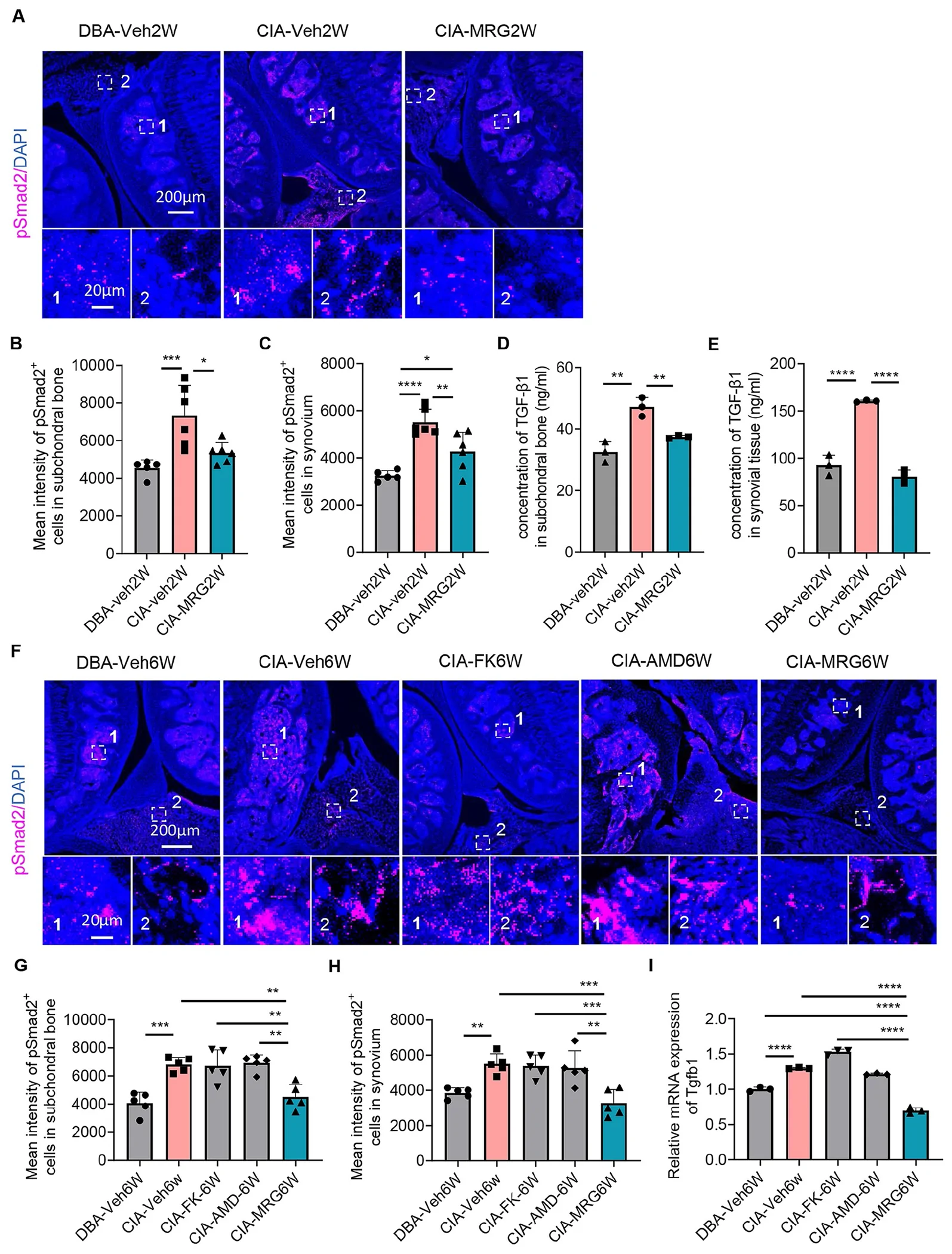
MRG-001 treatment inhibits activation of TGF-β signaling in CIA mice. (A–C) Representative images of pSmad2 IF staining in the tibial subchondral bone and synovium regions (A), with quantitative analysis of pSmad2-positive cells in the subchondral bone (B) and synovium (C) in DBA/1J mice and CIA mice treated with vehicle or MRG-001 for 2 weeks (*n* ≥ 6; one-way ANOVA with Tukey’s multiple comparisons test). (D, E) Quantitative analysis of TGF-β protein levels in subchondral bone (D) and synovial tissue (E) measured by ELISA in DBA/1J mice and CIA mice treated with vehicle or MRG-001 for 2 weeks (*n* = 3; one-way ANOVA with Tukey’s multiple comparisons test). (F–H) Representative images of pSmad2 immunofluorescence staining in the tibial subchondral bone and synovium regions (F), and quantitative analysis of pSmad2-positive cells in the subchondral bone (G) and synovium (H) in DBA/1J mice and CIA mice treated with vehicle, FK506, AMD3100, or MRG-001 for 6 weeks (*n* ≥ 5; one-way ANOVA with Tukey’s multiple comparisons test). (I) Quantitative analysis of TGF-β gene expression in knee joint tissue from DBA/1J mice and CIA mice treated with vehicle, FK506, AMD3100, or MRG-001 for 6 weeks (*n* ≥ 3; one-way ANOVA with Tukey’s multiple comparisons test). *P<0.05, **P<0.01, ***P<0.005, ****P<0.001.

Aberrant activation of TGF-β signaling in subchondral bone has been implicated in joint destruction in RA by disrupting normal bone remodeling and recruiting MSCs to pathological sites^[14]^. To evaluate whether MRG-001 modulates this signaling pathway, we measured the expression of phosphorylated Smad2 (pSmad2), a downstream effector of TGF-β signaling. IF staining revealed significantly elevated pSmad2 expression in the subchondral bone and synovium of saline-treated CIA mice after 2 weeks of treatment. This increase was not observed in MRG-001-treated mice (Fig. 4A–C). Similarly, TGF-β protein levels measured by ELISA were elevated in saline-treated CIA mice but remained near baseline in MRG-001-treated mice (Fig. 4D–E). At 6 weeks post-treatment, pSmad2 expression persisted in the joints of saline- and single-agent-treated CIA mice, whereas MRG-001-treated mice exhibited pSmad2 expression levels comparable to non-immunized controls (Fig. 4F–H). In parallel, TGF-β mRNA expression in joint tissue was significantly higher in saline- and single-agent-treated groups but was markedly reduced with MRG-001 treatment (Fig. 4I). These findings suggest that MRG-001 treatment may modulate aberrant TGF-β signaling in the subchondral bone and synovium.

**Figure 5.**
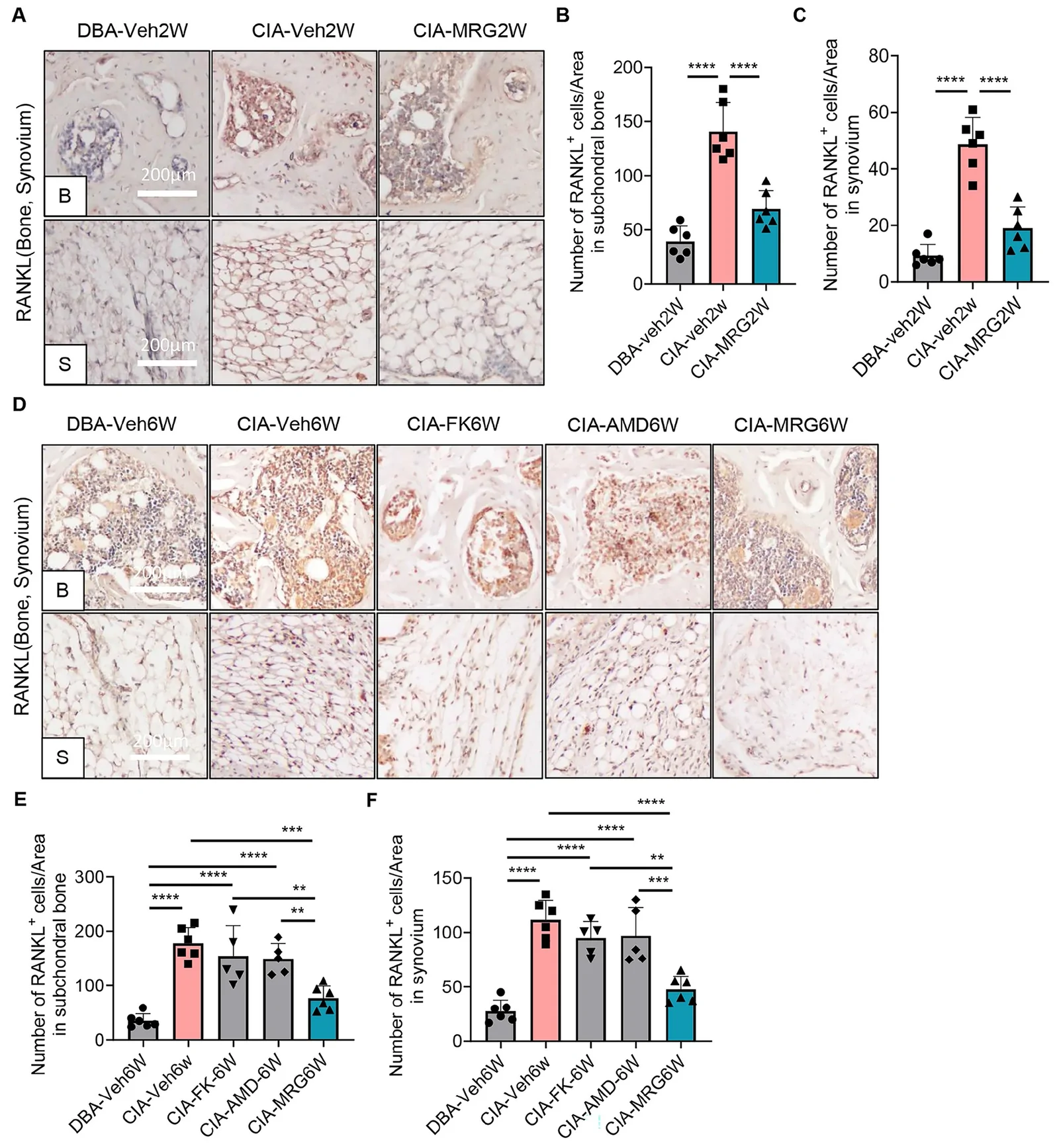
MRG-001 treatment reduces RANKL expression in CIA mice. (A–C) Representative images of RANKL IHC staining in the tibial subchondral bone region (A, upper row) and synovium region (A, lower row), along with quantitative analysis of RANKL-positive staining in the subchondral bone (B) and synovium (C) in DBA/1J mice and CIA mice treated with vehicle or MRG-001 for 2 weeks (*n* ≥ 6; one-way ANOVA with Tukey’s multiple comparisons test). B: Bone region; S: Synovium region. (D–F) Representative images of RANKL IHC staining in the tibial subchondral bone region (D, upper row) and synovium region (D, lower row), and quantitative analysis of RANKL expression in the subchondral bone (E) and synovium (F) in DBA/1J mice and CIA mice treated with vehicle, FK506, AMD3100, or MRG-001 for 6 weeks (*n* ≥ 5; one-way ANOVA with Tukey’s multiple comparisons test). B: Bone region; S: Synovium region. **P<0.01, ***P<0.005, ****P<0.001.

RANKL, primarily produced by FLSs, promotes osteoclast differentiation and activation, leading to bone resorption in RA^[18]^. IHC analysis demonstrated a significant increase in RANKL^+^ cells within the subchondral bone and synovium of saline-treated CIA mice at both 2 and 6 weeks. Single-agent treatment did not alter RANKL expression (Fig. 5E and F), whereas MRG-001-treated mice did not show the same increase at either time point (Fig. 5A–F). Together, these results suggest that MRG-001 may reduce FLS activation and is associated with lower levels of TGF-β signaling and RANKL expression, which could contribute to joint protection in CIA mice.

### MRG-001 treatment is associated with reduced abnormal angiogenesis in the knee joints of CIA mice

**Figure 6.**
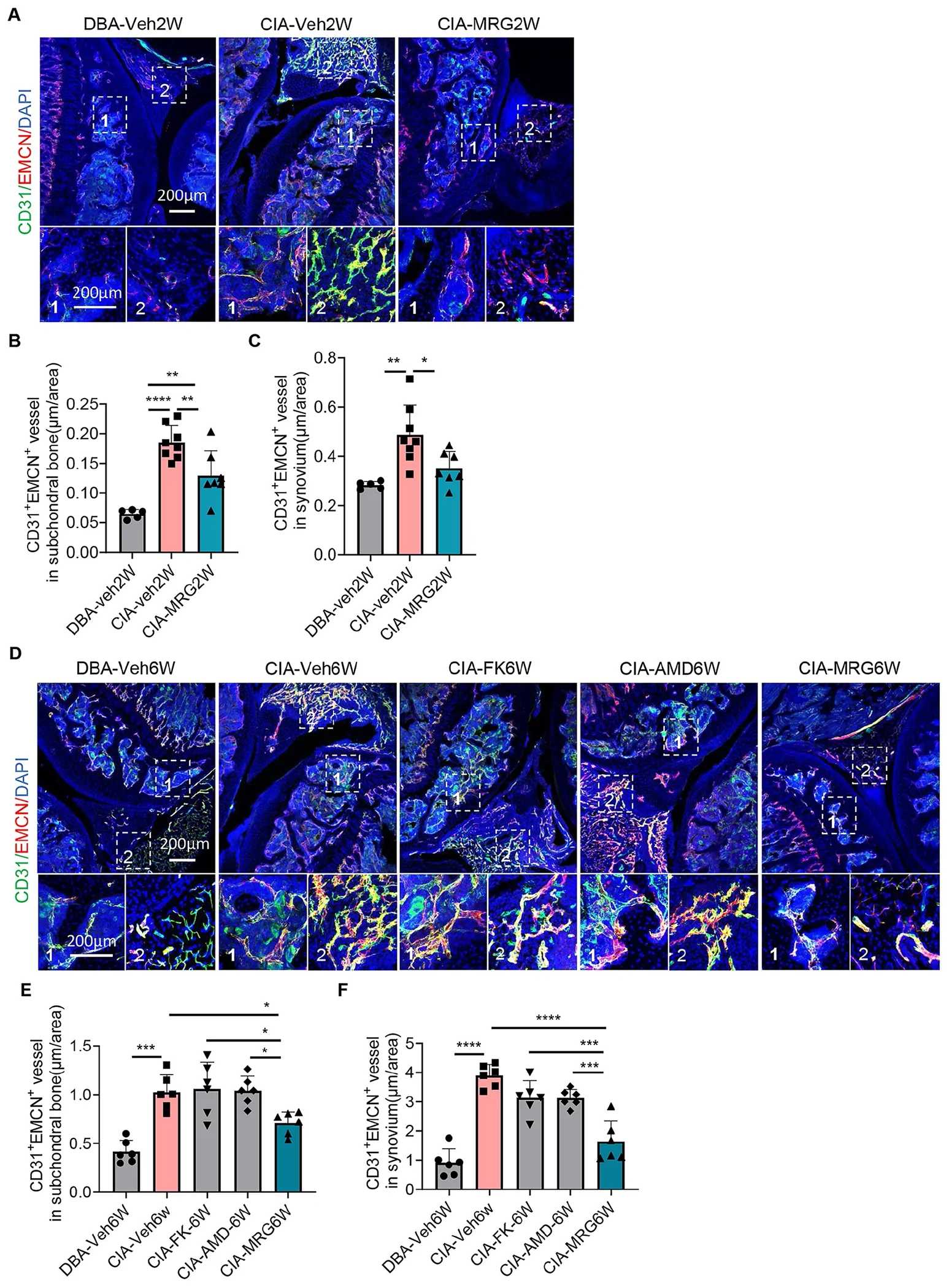
MRG-001 treatment prevents abnormal angiogenesis in the knee joints of CIA mice. (A–C) Representative images of CD31 and endomucin (EMCN) co-immunofluorescence staining in the tibial subchondral bone and synovium regions (A), with quantitative analysis of CD31^+^EMCN^+^ vessel density in the subchondral bone (B) and synovium (C) of DBA/1J mice and CIA mice treated with vehicle or MRG-001 for 2 weeks (*n* ≥ 5; one-way ANOVA with Tukey’s multiple comparisons test). (D–F) Representative images of CD31 and EMCN co-immunofluorescence staining in the tibial subchondral bone and synovium regions (D), and quantitative analysis of CD31^+^EMCN^+^ vessel density in the subchondral bone (E) and synovium (F) of DBA/1J mice and CIA mice treated with vehicle, FK506, AMD3100, or MRG-001 for 6 weeks (*n* = 6; one-way ANOVA with Tukey’s multiple comparisons test). *P<0.05, **P<0.01, ***P<0.005, ****P<0.001.

Abnormal angiogenesis – the pathological formation of new blood vessels – is a key contributor to RA pathogenesis, promoting pannus formation, inflammation, and joint destruction^[14,50– 52]^. This process is driven by multiple pro-angiogenic factors, and previous studies have shown that TGF-β inhibition suppresses abnormal angiogenesis in the subchondral bone of CIA mice^[14]^. To assess the effects of MRG-001 on pathological neovascularization in CIA joints, we examined CD31^+^EMCN^+^ type H vessels using IF double staining^[53]^. Compared to healthy controls, saline-treated CIA mice showed a marked increase in type H vessel density in both the subchondral bone and synovium at 2 weeks post-treatment (Fig. 6A–C), with further increases observed at 6 weeks (Fig. 6D–F). Treatment with either AMD3100 or FK506 alone did not reduce CD31^+^EMCN^+^ vessel formation. In contrast, MRG-001-treated mice showed significantly fewer type H vessels in both the subchondral bone (Fig. 6B, E) and synovium (Fig. 6C, F), suggesting a reduction in abnormal angiogenesis.

### MRG-001 treatment is associated with reduced aberrant sensory nerve innervation in the knee joints of CIA mice

**Figure 7.**
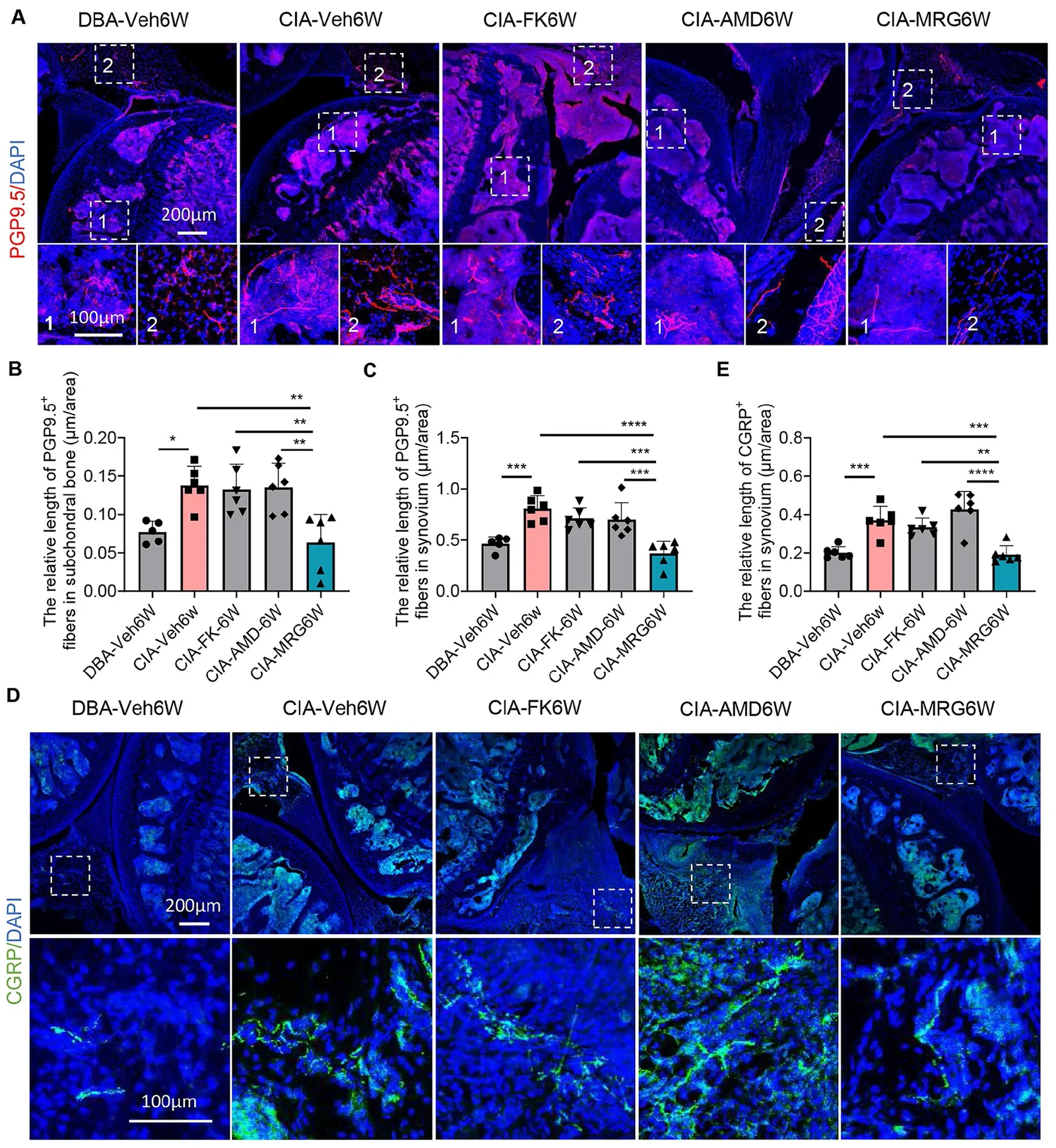
MRG-001 treatment eliminates aberrant nerve innervation in the knee joints. (A) Representative images of PGP9.5 immunofluorescence staining in the tibial subchondral bone and synovium regions. (B, C) Quantitative analysis of PGP9.5-positive nerve fiber density in the tibial subchondral bone (B) and synovium (C) of DBA/1J mice and CIA mice treated with vehicle, FK506, AMD3100, or MRG-001 for 6 weeks (*n* = 5; one-way ANOVA with Tukey’s multiple comparisons test). (D) Representative images of CGRP immunofluorescence staining in the tibial synovium region. (E) Quantitative analysis of CGRP-positive nerve fiber density in the tibial synovium region of DBA/1J mice and CIA mice treated with vehicle, FK506, AMD3100, or MRG-001 for 6 weeks (*n* ≥ 5; one-way ANOVA with Tukey’s multiple comparisons test). *P<0.05, **P<0.01, ***P<0.005, ****P<0.001.

Aberrant innervation by calcitonin gene-related peptide (CGRP)-positive sensory nerve fibers contributes to pain in RA^[54,55]^. To examine whether MRG-001 treatment is associated with changes in sensory nerve innervation, we evaluated the expression of PGP9.5^+^ (a pan-neuronal marker) and CGRP^+^ (a nociceptor marker) nerve fibers in the knee joints using IF staining. At 2 weeks post-treatment, the length of both PGP9.5^+^ and CGRP^+^ fibers was markedly increased in the subchondral bone and synovium of saline-treated CIA mice compared to nonimmunized healthy DBA mice (Supplemental Digital Content Figure 3, available at: http://links.lww.com/JS9/H347). This aberrant innervation persisted at 6 weeks post-treatment (Fig. 7A, B). MRG-001 treatment was associated with a significant reduction in PGP9.5^+^ and CGRP^+^ fiber presence in the subchondral bone and synovium at 2 weeks (Supplemental Digital Content Figure 3, available at: http://links.lww.com/JS9/H347). By 6 weeks post-treatment, nerve fiber density in MRG-001-treated CIA mice approached levels observed in non-immunized controls (Fig. 7C–E). These findings suggest that MRG-001 treatment is associated with reduced aberrant sensory nerve innervation, particularly CGRP^+^ fibers, which may be related to the observed improvement in pain-like behaviors in CIA mice.

### MRG-001 treatment is associated with reduced bone erosion, joint inflammation, and pain-like behaviors in a rapid-onset, antibody-mediated arthritis model

**Figure 8.**
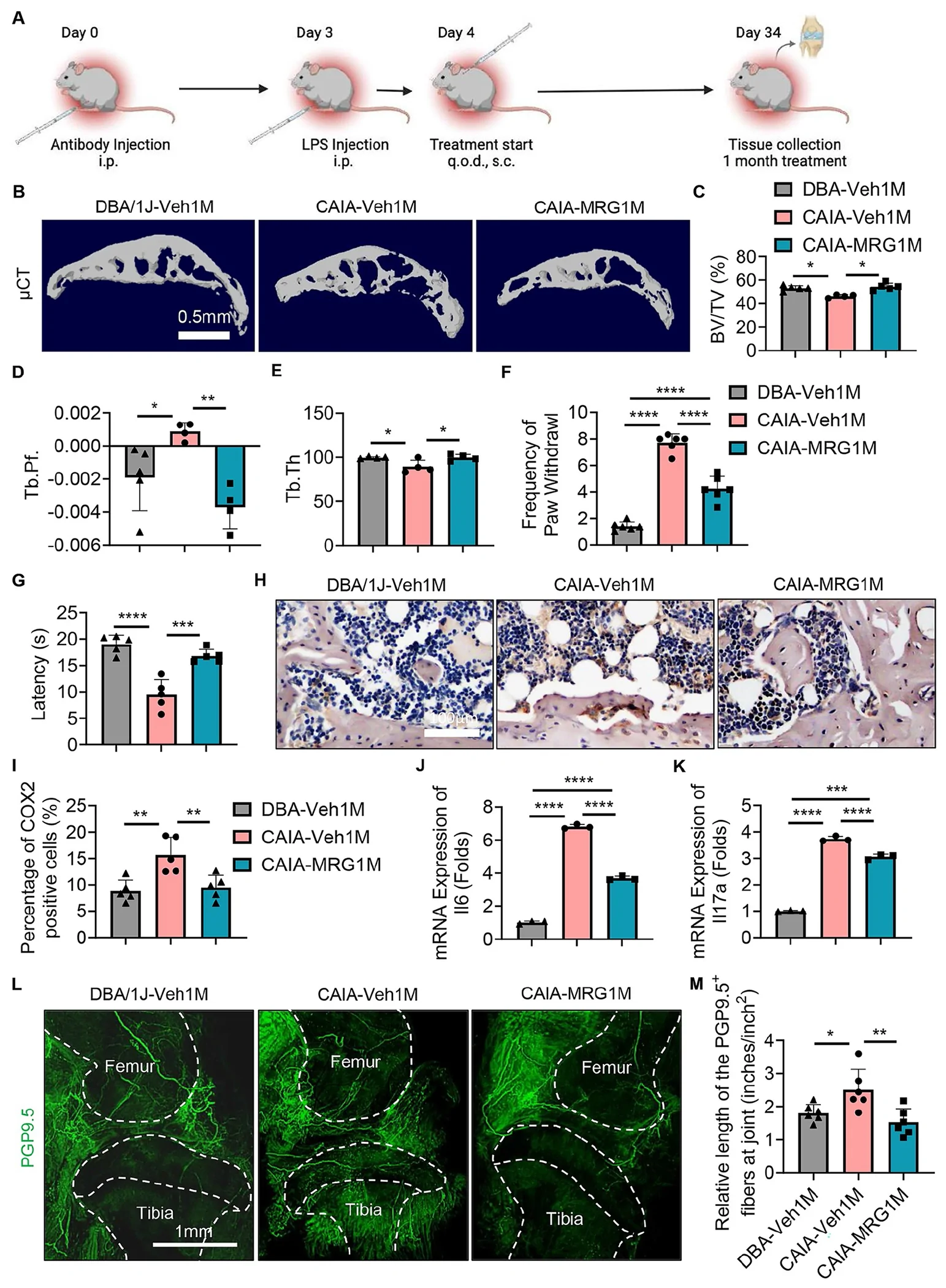
MRG-001 treatment prevents bone erosion, reduces inflammation, and alleviates pain-related behavior in the CAIA model. (A) Schematic diagram of the experimental timeline. (B) Representative 3D microCT images (sagittal view) of the subchondral bone in the medial tibial compartment from DBA/1J mice and CAIA mice treated with vehicle or MRG-001 for 1 month. (C–E) Quantitative analysis of microCT parameters including bone volume per tissue volume (BV/TV) (C), trabecular bone pattern factor (Tb.Pf) (D), and trabecular thickness (Tb.Th) (E) in DBA/1J and CAIA mice treated with vehicle or MRG-001 (*n* ≥ 4; one-way ANOVA with Tukey’s multiple comparisons test). (F, G) Behavioral assessment of pain-related responses, including mechanical hypersensitivity (paw withdrawal frequency, F) and thermal hypersensitivity (paw withdrawal latency, G), in DBA/1J and CAIA mice treated with vehicle or MRG-001 (*n* ≥ 5; one-way ANOVA with Tukey’s multiple comparisons test). (H, I) Representative images of COX-2 IHC staining in the tibial subchondral bone (H), and quantitative analysis of COX-2 expression (I) in DBA/1J and CAIA mice treated with vehicle or MRG-001 (*n* = 5; one-way ANOVA with Tukey’s multiple comparisons test). (J, K) Quantitative PCR analysis of IL-6 (J) and IL-17a (K) genes expression in knee joint tissue from DBA/1J mice and CAIA mice treated with vehicle or MRG-001 for 1 month (*n* = 3; one-way ANOVA with Tukey’s multiple comparisons test). (L, M) Representative images of PGP9.5 immunofluorescence staining in knee joints (L) and quantitative analysis of PGP9.5-positive nerve fibers (M) in DBA/1J and CAIA mice treated with vehicle or MRG-001 (*n* = 6; one-way ANOVA with Tukey’s multiple comparisons test). *P<0.05, **P<0.01, ***P<0.005, ****P<0.001.

To further evaluate the therapeutic efficacy of MRG-001 in RA, we utilized a more aggressive model – the CAIA model – which features faster disease onset and higher incidence. CAIA was induced by intraperitoneal injection of monoclonal antibodies against type II collagen (1.5 mg/mouse), followed by lipopolysaccharide (LPS; 25 μg/mouse) on day 3 to enhance disease severity^[37]^. Mice were randomly assigned to receive either placebo (saline) or MRG-001, administered every other day starting on day 4 post-antibody injection for one month (Fig. 8A).

Micro-CT analysis revealed significant reductions in bone volume and trabecular bone thickness (Tb.Th), as well as increased trabecular bone pattern factor (Tb.Pf) in saline-treated CAIA mice compared to non-immunized controls (DBA-Veh) (Fig. 8, B–E). MRG-001-treated mice exhibited bone volume, Tb.Th, and Tb.Pf values approaching those of healthy controls, suggesting an association with reduced bone erosion. Assessment of pain-like behaviors showed that saline-treated CAIA mice exhibited an increased paw withdrawal frequency and decreased latency in the Von Frey and Hargreaves tests, respectively – both indicative of heightened pain sensitivity. MRG-001 treatment was associated with reduced paw withdrawal frequency and increased withdrawal latency toward near-normal levels (Fig. 8F, G). COX-2 (cyclooxygenase-2), a key enzyme involved in prostaglandin synthesis, plays a critical role in inflammation, pain, and swelling in RA^[56]^. Immunohistochemistry revealed marked upregulation of COX-2 in the joints of saline-treated CAIA mice, whereas COX-2 expression was lower in MRG-001-treated mice (Fig. 8H, I). Furthermore, mRNA levels of the pro-inflammatory cytokines IL-6 and IL-17a were significantly elevated in joints of saline-treated mice but were reduced in MRG-001-treated mice (Fig. 8J, K).

In addition, whole-mount staining of knee joints revealed a significant increase in PGP9.5^+^ nerve fiber density – indicative of aberrant innervation – in saline-treated CAIA mice, whereas MRG-001-treated mice-maintained baseline innervation levels comparable to healthy controls (Fig. 8L, M). Taken together, these findings suggest that MRG-001 treatment is associated with reduced inflammation, preservation of bone structure, and improvement in pain-related behaviors in the CAIA model.

## Discussion

Despite advances in DMARDs, many RA patients experience inadequate responses or adverse effects, highlighting the need for novel therapies that address both immune dysregulation and structural joint damage. In this study, we show that MRG-001 is associated with multi-faceted therapeutic benefits in both the CIA and CAIA mouse models of RA.

MRG-001 treatment was associated with increased circulating CD45^−^Sca1^+^CD29^+^ MSCs, reduced CD4^+^RORγt^+^ Th17 cells, and increased CD4^+^Foxp3^+^ regulatory T cells in peripheral blood. These effects likely contribute to the resolution of systemic inflammation and restoration of immune homeostasis. MRG-001 also significantly reduced joint swelling, clinical arthritis scores, and the expression of key inflammatory cytokines, including TNF-α, IL-6, and IL-17a, all of which are well-established drivers of RA pathogenesis.

Beyond immunological regulation, MRG-001 markedly protected joint structure. Histological and micro-CT analyses revealed that MRG-001 prevented cartilage degradation, suppressed TRAP^+^ osteoclast activity, and reversed bone loss. Importantly, MRG-001 preserved trabecular bone architecture over time, restoring bone volume and parameters such as Tb.Th, Tb.N, Tb.Sp, and Tb.Pf to levels comparable to healthy controls. These findings indicate that MRG-001 halts bone erosion and promotes bone preservation, a critical unmet need in RA therapy.

MRG-001 treatment was associated with reduced markers of FLS activation, a major contributor to synovial hyperplasia and joint degradation. Treatment was associated with fewer vimentin^+^ FLSs and lower levels of TGF-β signaling markers, as evidenced by reduced pSmad2 expression and TGF-β mRNA/protein levels in subchondral bone and synovium. Furthermore, MRG-001 significantly decreased RANKL expression, a critical mediator of osteoclastogenesis and bone destruction, which may contribute to the observed preservation of bone integrity.

Notably, MRG-001 also targets two non-classical yet clinically significant pathological features of RA: aberrant angiogenesis and nerve innervation. Our previous study demonstrated that the abnormal distribution of type H vessels is associated with uncoupled bone remodeling and contributes to joint erosion in RA mice^[14]^. MRG-001 treatment was associated with fewer CD31^+^EMCN^+^ type H vessels in both the subchondral bone and synovium, potentially related to changes in TGF-β signaling activity – consistent with previous reports implicating TGF-β in pathological neovascularization^[14]^.

Additionally, MRG-001 treatment was associated with reduced aberrant innervation of PGP9.5^+^ and CGRP^+^ sensory nerve fibers, which are linked to RA-associated pain^[54,55]^. These findings are consistent with the behavioral improvements observed in pain assays, including thermal and mechanical hyperalgesia, as well as voluntary wheel-running activity.

Importantly, the therapeutic effects of MRG-001 were validated in the CAIA model, which mimics the effector phase of RA and is characterized by rapid disease onset and robust joint damage driven by autoantibodies. In this model, MRG-001 reduced joint inflammation, improved pain-like behaviors, prevented bone erosion, and lower expression of pro-inflammatory mediators including COX-2, IL-6, and IL-17a. These results underscore the broad and reproducible efficacy of MRG-001 across multiple RA models.

The individual components of MRG-001 – FK506 and AMD3100 – exhibited minimal or no effect on RA pathogenesis when administered alone. This finding is consistent with previous studies in wound healing^[23,25,27]^ and transplantation models^[22,24,29]^, which demonstrated that the combination of AMD3100 and low-dose FK506 exerts synergistic effects on inflammation resolution and tissue repair/regeneration. Tacrolimus, an immunosuppressive agent, has been used in RA treatment by inhibiting T cell activation^[57]^. However, the dose of FK506 in MRG-001 is less than one-tenth of the conventional immunosuppressive dosage and, on its own, does not impact RA progression. This supports the notion that MRG-001’s therapeutic efficacy is not dependent on the immunosuppressive properties of Tacrolimus, but rather on a non-immunosuppressive mechanism^[58]^ that contributes to its anti-inflammatory and regenerative effects. However, it is important to note that the current data primarily demonstrate associations between MRG-001 treatment and downstream biological outcomes. The precise causal mechanisms remain to be elucidated in future studies. Also, this study cannot distinguish between synergistic and dose-dependent effects^[59,60]^.

While MRG-001 demonstrated promising anti-inflammatory and bone-protective efficacy in RA models, its clinical translation requires careful consideration of long-term safety. Notably, the FK506 dose incorporated into MRG-001 is non-immunosuppressive, with no detectable systemic trough levels and no evidence of drug accumulation. Comprehensive GLP-compliant toxicity studies in rodents and swine have established a favorable safety margin (U.S. Patent No. 12,115,149). Nonetheless, the potential risks associated with repeated stem cell mobilization warrant ongoing evaluation. Future clinical studies should incorporate longitudinal immunological monitoring, surveillance for hematopoietic dysregulation, and tumorigenicity assessments to ensure sustained safety and minimize immune interference.

The present study focused on evaluating the therapeutic efficacy of MRG-001 in treating RA, rather than dissecting its precise molecular mechanisms. MRG-001 consistently demonstrated protective effects on joint integrity and pain relief in two distinct RA mouse models, providing strong preclinical support for further development. Given its broad effects on immune modulation, angiogenesis, innervation, and tissue remodeling, MRG-001 likely exerts its action through complex and multifactorial pathways. Future mechanistic investigations, such as cell-type specific interventions, pathway inhibition studies, or genetic knockout models, will be necessary to clarify the molecular underpinnings and causal relationships behind the observed therapeutic benefits.

## Conclusion

Taken together, our findings support the potential of MRG-001 as a promising multi-targeted therapeutic candidate for RA. Its ability to simultaneously modulate systemic immune responses and preserve local joint architecture distinguishes it from conventional therapies that primarily focus on single inflammatory mediators. Moving forward, clinical studies are warranted to evaluate the long-term safety, immunological tolerance, and therapeutic efficacy of MRG-001, both as a monotherapy and in combination with existing DMARDs.

## Supplementary Material

Supplemental Figure 1

Supplemental Figure 2

Supplemental Figure 3

supplementary figure legends

Supplemental Digital Content is available for this article. Direct URL citations are provided in the HTML and PDF versions of this article on the journal’s website, www.lww.com/international-journal-of-surgery.

## Data Availability

All the data related to this study presented in the manuscript.

## HIGHLIGHTS

- MRG-001 shows strong therapeutic effects in rheumatoid arthritis preclinical models.
- It reduces inflammation and mobilizes mesenchymal stem cells.
- MRG-001 restores immune balance and protects cartilage and bone.
- It inhibits aberrant TGF-β signaling and pathological angiogenesis.
- MRG-001 reduces sensory innervation, improving joint function and pain.
