# Supplementary material for: Mobilizing stem cells and inhibition of autoimmunity synergistically modify autoimmune disease to relieve pain: a potential MRG-001 therapy for rheumatoid arthritis
Source: Int J Surg. Author manuscript; Available in PMC 2026 Sep 3. (PMC13537757; doi:10.1097/js9.0000000000005353)
Supplement: Supplementary file 1 [file NIHMS2182253-supplement-supplementary_figure_legends.docx]

**Supplemental Figure 1. MRG-001 treatment mobilizes CD45⁻Sca1⁺CD29⁺ mesenchymal stem cells, decreases CD4⁺RORγt⁺ Th17 cells, and restores CD4⁺Foxp3⁺ regulatory T cells in the peripheral blood of CIA mice.**

(**A-C**) Quantitative analysis of peripheral blood cell populations in DBA/1J and CIA mice treated with vehicle or MRG-001 for 2 weeks: mesenchymal stem cells (CD45⁻Sca1⁺CD29⁺) (A), Th17 cells (CD4⁺RORγt⁺) (B), and regulatory T cells (CD4⁺CD25⁺Foxp3⁺) (C). (n ≥ 3; one-way ANOVA with Tukey’s multiple comparisons test). (**D**, **E**) Quantitative analysis of Th17 cells (CD4⁺RORγt⁺) (D) and regulatory T cells (CD4⁺CD25⁺Foxp3⁺) (E) in peripheral blood from DBA/1J and CIA mice treated with vehicle, FK506, AMD3100, or MRG-001 for 6 weeks. (n ≥ 3; one-way ANOVA with Tukey’s multiple comparisons test). *: *P*＜0.05, **: *P*＜0.01, ***: *P*＜0.005.

**Supplemental Figure 2. MRG-001 improves bone structure in the CIA model two weeks after treatment.**

CIA mice were treated with vehicle or MRG-001 for 2 weeks; vehicle-treated healthy DBA/1J mice served as controls. (**A**) Quantitative analysis of bone volume/tissue volume (BV/TV). (**B**) Quantitative analysis of trabecular bone pattern factor (Tb.Pf). (**C**) Quantitative analysis of trabecular thickness (Tb.Th). (**D**) Quantitative analysis of trabecular number (Tb.N). (**E**) Quantitative analysis of trabecular separation (Tb.Sp). (n ≥ 7; one-way ANOVA with Tukey’s multiple comparisons test). *: *P*＜0.05, **: *P*＜0.01.

**Supplemental Figure 3. MRG-001 eliminates aberrant nerve innervation in the knee joints two weeks after treatment.**

(**A**-**C**) Representative images of PGP9.5 immunofluorescence staining in the tibial subchondral bone and synovium (A), and quantitative analysis of PGP9.5 staining in the tibial subchondral bone (B) and synovium (C) in DBA/1J mice and CIA mice treated with vehicle or MRG-001 for 2 weeks. (n = 5; one-way ANOVA with Tukey’s multiple comparisons test). (**D**, **E**) Representative images of CGRP immunofluorescence staining in the tibial synovium (D), and quantitative analysis of CGRP staining (E) in DBA/1J and CIA mice treated with vehicle or MRG-001 for 2 weeks. (n ≥ 5; one-way ANOVA with Tukey’s multiple comparisons test). *: *P*＜0.05, **: *P*＜0.01, ***: *P*＜0.005, ****: *P*＜0.001.
